# PDX models of human lung squamous cell carcinoma: consideration of factors in preclinical and co-clinical applications

**DOI:** 10.1186/s12967-020-02473-y

**Published:** 2020-08-06

**Authors:** Hae-Yun Jung, Tae Ho Kim, Jong-Eun Lee, Hong Kwan Kim, Jong Ho Cho, Yong Soo Choi, Sumin Shin, Se-Hoon Lee, Hwanseok Rhee, Hee Kyung Lee, Hyun Jung Choi, Hye Yoon Jang, Seungjae Lee, Jung Hee Kang, Young Ae Choi, Sanghyuk Lee, Jinseon Lee, Yoon La Choi, Jhingook Kim

**Affiliations:** 1 Department of Thoracic and Cardiovascular Surgery, Samsung Medical Center, Sungkyunkwan University School of Medicine, Seoul, South Korea; 2 DNA Link, Inc, Seoul, South Korea; 3 Division of Hematology-Oncology, Department of Medicine, Sungkyunkwan University School of Medicine, Seoul, South Korea; 4 Ewha Research Center for Systems Biology (ERCSB) and Department of Life Science, Ewha Womans University, Seoul, South Korea; 5 Department of Pathology, Samsung Medical Center, Sungkyunkwan University School of Medicine, Seoul, South Korea

**Keywords:** Lung squamous cell carcinoma, Patient-derived xenograft, Engraftment, Preclinical model, Xenograft-associated lymphoproliferative disease

## Abstract

**Background:**

Treatment of human lung squamous cell carcinoma (LUSC) using current targeted therapies is limited because of their diverse somatic mutations without any specific dominant driver mutations. These mutational diversities preventing the use of common targeted therapies or the combination of available therapeutic modalities would require a preclinical animal model of this tumor to acquire improved clinical responses. Patient-derived xenograft (PDX) models have been recognized as a potentially useful preclinical model for personalized precision medicine. However, whether the use of LUSC PDX models would be appropriate enough for clinical application is still controversial.

**Methods:**

In the process of developing PDX models from Korean patients with LUSC, the authors investigated the factors influencing the successful initial engraftment of tumors in NOD scid gamma mice and the retainability of the pathological and genomic characteristics of the parental patient tumors in PDX tumors.

**Conclusions:**

The authors have developed 62 LUSC PDX models that retained the pathological and genomic features of parental patient tumors, which could be used in preclinical and co-clinical studies.

*Trial registration* Tumor samples were obtained from 139 patients with LUSC between November 2014 and January 2019. All the patients provided signed informed consents. This study was approved by the institutional review board (IRB) of Samsung Medical Center (2018-03-110)

## Introduction

Lung cancer is one of the main causes of cancer-related death worldwide including Korea [1, 2]. Lung cancer is histopathologically categorized into non-small cell lung cancer (NSCLC) and small cell lung cancer (SCLC). The most common subtypes of NSCLC are lung adenocarcinoma (LUAD) and lung squamous cell carcinoma (LUSC), which have been extensively studied at the molecular level, revealing significant differences in somatic mutation profiles between those two subtypes [3–7]. LUAD has frequent driver mutations in *EGFR* and *ALK*, which serve as molecular targets for tyrosine kinase inhibitor-targeted therapy [8–10]; whereas, LUSC has diverse mutation profiles without any specific dominant mutations and has been rendered unresponsive to molecularly targeted drugs currently available [11, 12]. Therefore, more effective therapeutic strategies such as the combination of chemotherapy and targeted therapy would be needed for the treatment of LUSC, and thus, clinically relevant preclinical models would be required and validated for this purpose.

Patient-derived xenograft (PDX) models have been introduced to overcome the limitation of conventional preclinical models [13, 14]. PDX models permit us to expand the limited patient tumor materials, while retaining the characteristics, such as tumor heterogeneity and tumor microenvironment to some extent, of the parental patient tumors, which could give the ample opportunity for biomarker and drug discovery and treatment regimens [14, 15]. A substantial number of reports have suggested that PDX models could be used as preclinical study tools either for drug efficacy testing or for the development of new therapeutic strategies [16–19]. However, the potential utility of PDX models in preclinical or co-clinical studies has not been extensively studied at a large scale [20–22]. In particular, PDX models for LUSC with poor prognosis when current targeted therapies are used could be considered as unmet medical needs.

In this study, the authors studied the factors influencing the initial engraftment of tumors in NOD scid gamma (NSG) mice from 62 Korean patients with LUSC, which would be critical for the successful generation of the appropriate preclinical models for individual patients. The authors then examined how extensive LUSC PDX models can retain the characteristics of their parental patient tumors at the levels of histopathology and genetic and transcriptomic profiles. The data in this study supported that LUSC PDX models could be used as preclinical study models for drug discovery and the development of new therapeutic strategies.

## Materials and methods

### Tumor samples from patients with LUSC

Tumor samples were obtained from 139 patients with LUSC between November 2014 and January 2019. All the patients provided signed informed consents. This study was approved by the institutional review board (IRB) of Samsung Medical Center (2018-03-110). Clinical features of studied patients with LUSC are summarized in Additional file 1. Table S1. Clinical data such as age, gender, smoking status, stage, tumor size, preoperative chemotherapy, differentiation, vascular invasion, perineural invasion, lymphatic invasion, pleural invasion, recurrence, and survival were obtained from the patients’ medical records. Overall survival (OS) is defined as the time between histological diagnosis and death or the last follow-up, and relapse-free survival (RFS) is defined as the time between histological diagnosis and the first progression or recurrence, death as a result of disease, or the last follow-up.

### Establishment of LUSC PDX models

Tumor samples for patients with LUSC were subcutaneously implanted into the flanks of NSG mice (Jackson Laboratory, Sacramento, CA, USA) to establish PDX models. Once the tumor reached 60 mm^3^ in volume, its size was measured by a caliper twice a week. The tumor volume was calculated as 0.5 × length × width^2^. When the tumor size reached 800–1000 mm^3^, the mice were euthanized and the tumors were harvested for subsequent studies such as successive passaging of the PDX model, next generation sequencing analysis, and preparation of formalin-fixed paraffin-embedded blocks, and were stored as snap frozen tumor fragments. All animal care and experiments were performed under an animal protocol that had been approved by the Biomedical Research Institute at Seoul National University Hospital.

### Whole exome sequencing (WES)

Genomic DNA (3 μg) was used for constructing DNA libraries. To generate exome sequencing libraries, target enrichment was performed using the Agilent SureSelect Human All Exon V3 kit (Agilent Technologies, Inc., Santa Clara, CA, USA), according to the manufacturer’s instructions. Exon capturing was performed using the Agilent SureSelect 50 Mb system, and paired-end DNA sequences were obtained with the Illumina sequencing system HiSeq 2000 (Illumina Inc., San Diego, CA, USA). The sequenced reads were aligned to the University of California Santa Cruz hg19 release of the human genome. Somatic mutations were identified using MuTect, VarScan 2, and the GATK Somatic Indel Detector [23–25]. Selected mutations were verified using Sanger sequencing. Significantly mutated genes were identified with MutSigCV [26] and the functional enrichment of the somatic mutations was assessed using Metacore (GeneGo Inc., St. Joseph, MI, USA).

### Whole transcriptome sequencing (WTS)

mRNA libraries (insert size of ∼300 bp) were prepared using the TruSeq RNA Library Preparation Kit v2 (Illumina Inc., San Diego, CA, USA). The total RNA (1 μg) from each case sample was used to create the library, and samples were subjected to 101-bp paired-end sequencing on the Illumina sequencing system HiSeq 2000. Library preparation and sequencing procedures were performed at DNA Link, Inc.

### Pathological analysis

Patient and PDX tissue sections were freshly cut to 3 μm. Hematoxylin and eosin (H&E) staining was performed by an automatic machine called Symphony (Ventana Medical Systems, Inc., Roche, Basel, Switzerland), according to the manufacturer’s instructions. Immunohistochemical (IHC) staining for CK5, p63, TTF1, pan-cytokeratin, and CD105 was performed on a single representative block using the following procedures: Deparaffinized slides were treated with citrate buffer (pH 6.0) for antigen retrieval. Next, the primary antibody was incubated with the Dako antibody diluent (S3022, Dako, Agilent Technologies, Inc., Santa Clara, CA, USA) and then incubated with Dako REAL EnVision Detection System (K5007, Dako, Agilent Technologies, Inc., Santa Clara, CA, USA). The H&E and IHC images were analyzed on the ScanScope® XT scanner (Aperio, Leica Biosystems, Newcastle, UK). Antibody sources and dilution are summarized in Additional file 2. Table S2.

### EBV-encoded RNA in situ hybridization

Epstein–Barr virus (EBV) status was determined by EBV-encoded small RNA (EBER) in situ hybridization. The whole process was performed on a fully automatic system (BOND-MAX) using an EBV-encoded RNA probe from Leica Biosystems, according to the manufacturer’s instructions.

### Statistical analyses

The chi-squared or Fisher’s exact test was performed for comparisons between PDX engraftment status and patient characteristics. OS and RFS according to PDX engraftment status were assessed using the Kaplan–Meier curves and the log-rank test. To analyse clinically significant prognostic variables, the Cox proportional hazards model was used for multivariate analyses of OS and PFS. The Statistical Package for the Social Sciences software package version 25.0 (International Business Machines Corporation, Chicago, IL, USA) was used for all statistical analyses. A *p* value of < 0.05 was considered to indicate significance, and all *p*-values were two-sided.

## Results

### Factors affecting the success rate of initial engraftment

**Fig. 1:**
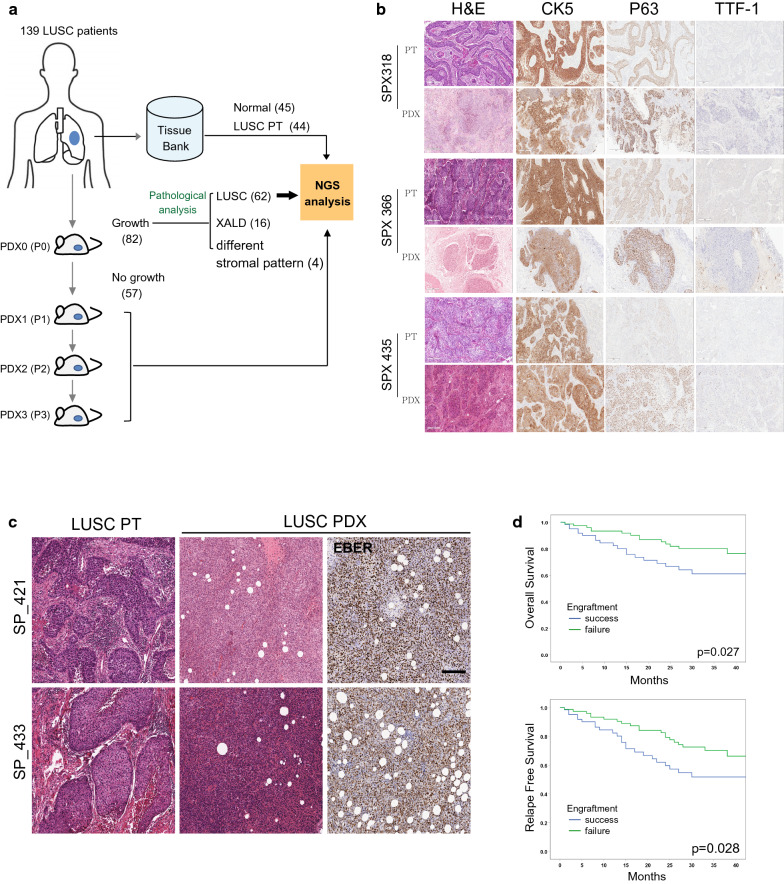
Establishment of PDX models from tumor samples from Korean patients with LUSC. **a** A schematic diagram of the experimental procedure for LUSC PDX models and the subsequent NGS analysis. **b** Representative histological and IHC-stained images of tumor samples from patients with LUSC and PDX models. Scale bars, 200 μm. **c** Representative histological images showing the tumor samples from the patients with LUSC and PDX models. Epstein–Barr virus (EBV)-encoded RNA (EBER) in situ hybridization images from PDX tumor samples. Brown indicates Epstein–Barr virus positive. Scale bars, 200 μm. **d** Kaplan–Meier plot showing overall survival (OS) or relapse-free survival (RFS) of the patients whose tumor engraftment was either successful or unsuccessful

A total of 139 patients with LUSC were enrolled between November 2014 and January 2019, and tumor samples from these patients were implanted into NSG mice to establish PDX models. Among them, 82 cases showed engraftment (59%), and 57 cases did not (41%) (Fig. 1a). On average, a period of 12.5 weeks was required for the initial successful engraftment of human tumor fragments in NSG mice. For the initial validation of the appropriateness of the PDX models, the authors checked for the pathological concordance between the tumors from the patients and the successfully engrafted PDX. Sixty-two cases showed the immunohistochemical staining patterns similar to their parental LUSC tumors, as determined by IHC with antibodies against human squamous cell carcinoma-specific proteins, CK5, p63, and TTF1 (three typical cases shown in Fig. 1b); whereas, 16 cases were found to have xenograft-associated lymphoproliferative diseases (XALDs), and 4 cases showed histologically different stromal patterns, compared to the parental patient tumors, which were even distinctive from most of the other PDX tumors for unknown reasons. For the XALD cases, some were found to be EBV-positive by RNA in situ hybridization (Fig. 1c) and others EBV-negative. However, the authors were not able to find any correlation between XALD incidence and the patients’ clinical parameters such as tumor stages, tumor sizes, and degree of differentiation (Additional file 3. Table S3).

Furthermore, the authors found no significant correlation between the success in the tumor engraftment and the clinical characteristics such as age, gender, smoking status, tumor stage, tumor size, degree of differentiation, and recurrence (Table 1). However, it was obvious that the failure in tumor engraftment was correlated with the patients’ experience with preoperative chemotherapy (*P* = 0.033; hazard ratio (HR) = 3.816; 95% confidence interval (CI) = 1.037–14.047), indicating that preoperative chemotherapy might have significantly lowered the number of viable tumor cells in the resected patient tumors. Furthermore, consistent with the results of previous reports [27, 28], the initial success of tumor engraftment into NSG mice was found to be correlated with OS (*P* = 0.027) or RFS (*P* = 0.028) (Fig. 1d), which suggests that the tumors from the patients with advanced LUSC were more likely to be engrafted at a higher rate.

**Table 1:** Clinical characteristics between successful and failed PDX engraftment

N = 139	PDX success	PDX failure	*p* value
Age(years) Mean	66.4 ± 6.8	64.8 ± 8.9	0.244*
Gender			
Male	60 (96.8%)	73 (94.8%)	0.692**
Female	2 (3.2%)	4(5.2%)	
Smoking status			
Former or current	61 (98.4%)	76 (98.7%)	1.000**
Never	1 (1.6%)	1 (1.3%)	
Preoperative Chemotherapy			
Yes	3 (4.8%)	13 (16.9%)	0.033**
No	59 (95.2%)	64 (83.1%)	
pTNM stage (7th)			
I	18 (29.0%)	28 (36.4%)	0.348**
II	29 (46.8%)	25 (32.5%)	
III	14 (22.6%)	23 (29.9%)	
IV	1 (1.6%)	1 (1.3%)	
Tumor size			
< 3 cm	13 (21.0%)	25 (32.5%)	0.204**
3 < < 5	21 (33.9%)	27 (35.1%)	
5 < < 7	21 (33.9%)	22 (28.6%)	
7 >	7 (11.3%)	3 (3.9%)	
Differentiation			
Well	1 (1.6%)	1 (1.1%)	1.000**
Moderate	47 (75.8%)	58 (76.3%)	
Poor	14 (22.6%)	17 (22.4%)	
Recurrence			
Yes	14 (22.6%)	14 (18.2%)	0.671**
No	48 (77.4%)	63 (81.8%)	
Vascular invasion			
Yes	8 (12.9%)	8 (10.4%)	0.399**
No	54 (87.1%)	69(92.2%)	
Perineural invasion			
Yes	8 (12.9%)	6 (7.8%)	0.399**
No	54 (87.1%)	71(92.2%)	
Lymphatic invasion			
Yes	22 (35.5%)	21 (27.3%)	0.357**
No	40 (64.5%)	56 (72.7%)	
Visceral pleural invasion			
PL0	46 (74.2%)	61 (81.3%)	0.599**
PL1	1 (1.6%)	2 (2.7%)	
PL2	6 (9.7%)	6 (8.0%)	
PL3	9 (14.5%)	6 (8.0%)	

The asterisks (*, **) indicate results from a Mann-Whitney U test and a Fisher’s exact test, respectively

### Comparison of genetic alterations and transcriptome profiling between the tumors of LUSC PDX models and patients

**Fig. 2:**
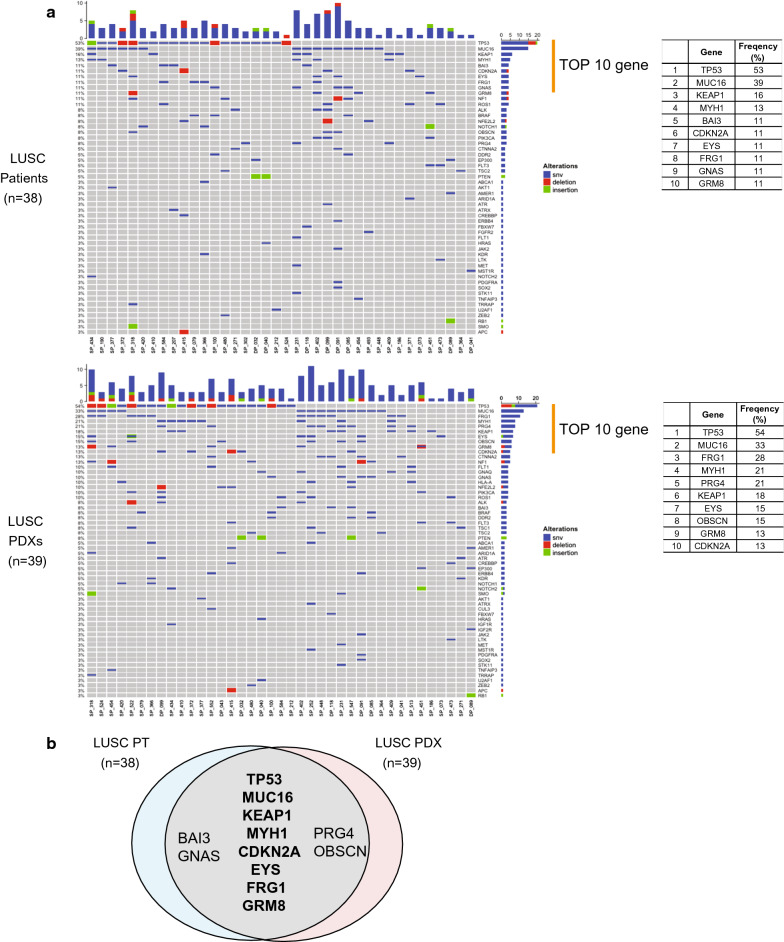
Frequency of somatic mutations in the tumor samples from the PDX models and patients with LUSC. **a** OncoPrint of somatic alterations in 38 patients with LUSC and 39 PDX models. The x-axis represents each sample ID and the y-axis represents the frequency of somatic mutations in the genes. The bar graph indicates the number of somatic alterations in each sample. The table indicates the 10 genes with the highest frequencies of somatic mutations. **b** The Venn diagram represents the shared genes of somatic mutations between the patients with LUSC and PDX models. Bold indicates the genes with the highest frequencies in both the patients and PDX models

The authors analyzed the WES of the tumor samples from patients with LUSC and LUSC PDX models for the preservation of genetic fidelity, one of the requirements of being a preclinical model for molecularly targeted therapy. The majority of the tumors in the PDX models had genetic alterations similar to those of their parental patient tumors (Additional file 4. Table S4). Higher frequencies of somatic mutation were observed in the *TP53*, *MUC16*, *KEAP1*, *MYH1*, *CDKN2A*, *EYS*, *FRG1*, and *GRM8* genes in both the PDX models and patients (Fig. 2a). However, the *BAI3*, *GNAS*, *PRG4*, and *OBSCN* genes showed different patterns of mutation frequency between PDX models and patients such that *BAI3* and *GNAS* genes showed relatively higher mutation frequencies in the tumor samples from the patients (11 and 11%, respectively) compared with those in the tumor samples from the PDX models (8 and 10%, respectively), and conversely *PRG4* and *OBSCN* genes showed higher mutation frequencies in the tumor samples from PDX models (21 and 15%, respectively) compared with those in the tumor samples from the patients (8 and 8%, respectively) (Fig. 2b).

**Fig. 3:**
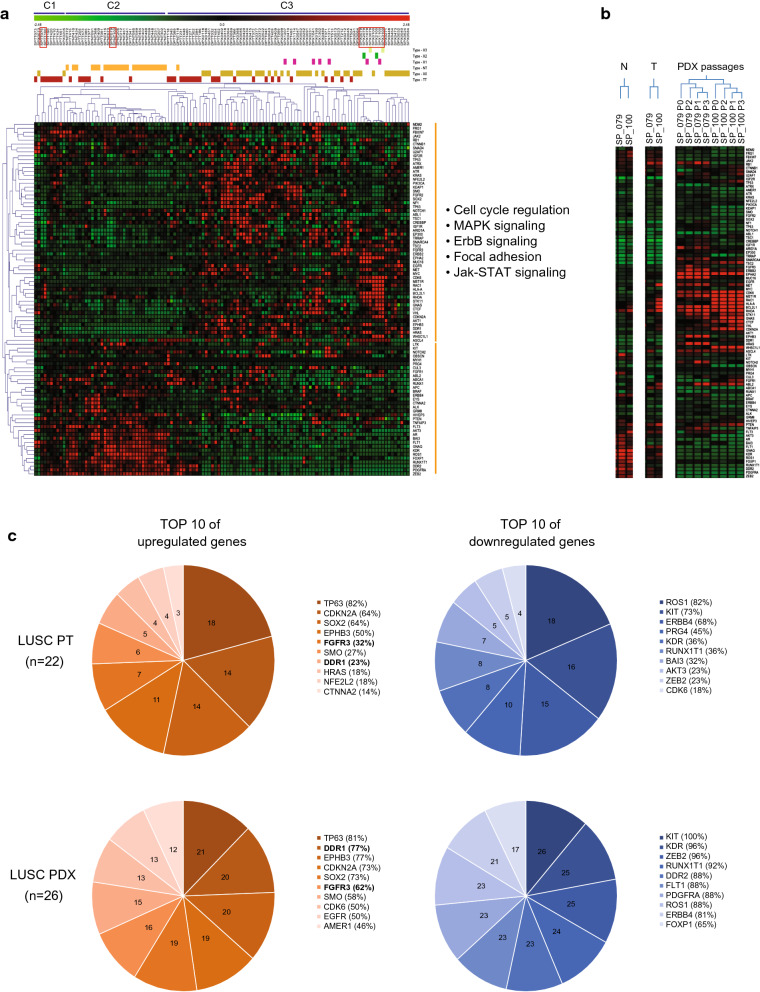
Gene expression patterns in normal individuals, patients with LUSC, and PDX models. **a** A heat map of gene expression patterns in the tissues samples from normal individuals, patients with LUSC, and PDX models. Clustering was made by sample types: C1 for the samples from the patients with LUSC, C2 for the normal tissue samples and several tumor samples from patients with LUSC, and C3 for the tumor samples from the patients with LUSC and PDX models. Orange indicates normal tissue samples, red indicates tumor samples, gold indicates PDX passage 0, pink indicates PDX passage 1, green indicates PDX passage 2, and light green indicates PDX passage 3. The signaling pathway was generated by functional annotation in the Database for Annotation, Visualization and Integrated Discovery (DAVID) website. **b** Heat map for SP_079 and SP_100 models in the red boxes from A. **c** DEGs between the patients with LUSC and PDX models. The Venn diagram represents the 10 most upregulated or downregulated genes in the patients with LUSC and PDX models

Next, the authors analyzed the WTS of the tumor samples from the LUSC PDX models and patients to determine whether they retain the gene expression fidelity. The tumor samples and samples of their adjacent normal tissues from patients with LUSC and PDX models showed remarkably different patterns of gene expression. The major tumor group C3 presented higher expressions of cell proliferation-related genes than the other tumor groups C1 and C2 (Fig. 3a). However, the authors found no visible clinical characteristics differentiating these three distinct groups, which had been derived from the gene clustering analysis (data not shown).

**Fig. 4:**
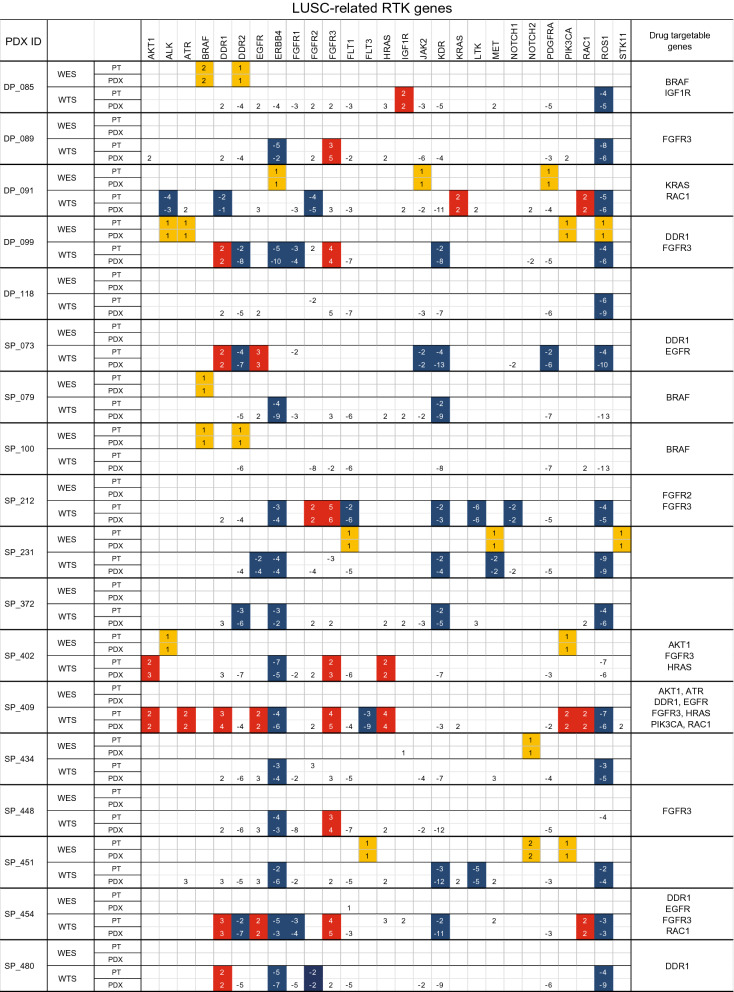
Distribution of genetic changes in several receptor tyrosine kinase genes of the patients with LUSC and PDX models. WES is the source for the somatic mutations of the genes and WTS for the DEGs. The yellow box indicates shared somatic mutations in the patients with LUSC and PDX models; the red box indicates upregulated genes in both the patients with LUSC and PDX models; and the blue box indicates downregulated genes in both the patients with LUSC and PDX models. The number represents the frequency of somatic mutations or fold changes of the gene

However, analysis of the differentially expressed genes (DEGs) in the tumor samples from the PDX models and patients with LUSC showed that those genes were congruent to each other (Fig. 3c), which could suggest that these DEGs are likely to be essential for tumor viability regardless of the different hosts, human or mice. The authors compared the exome and transcriptomic profiles between the patients and PDX models in terms of somatic mutations and DEGs (Fig. 4 and Additional file 5. Table S5). Interestingly, *FGFR3* was highly expressed in both the tumor samples from the PDX models and patients, implying a promising molecular target for LUSC, which had been suggested by other reports that either FGFR-targeted therapy alone or the combination of FGFR1-targeted therapy and chemotherapy could be beneficial in the treatment of LUSC [18, 22]. In the LUSC PDX models in this study, three PDX models, DP089, SP212, and SP448, which had upregulated expression of the *FGFR3* gene, could be used as preclinical models to test in vivo the efficacy of FGFR-targeted drugs.

### Pathological and genomic consistency during PDX tumor passages

**Fig. 5:**
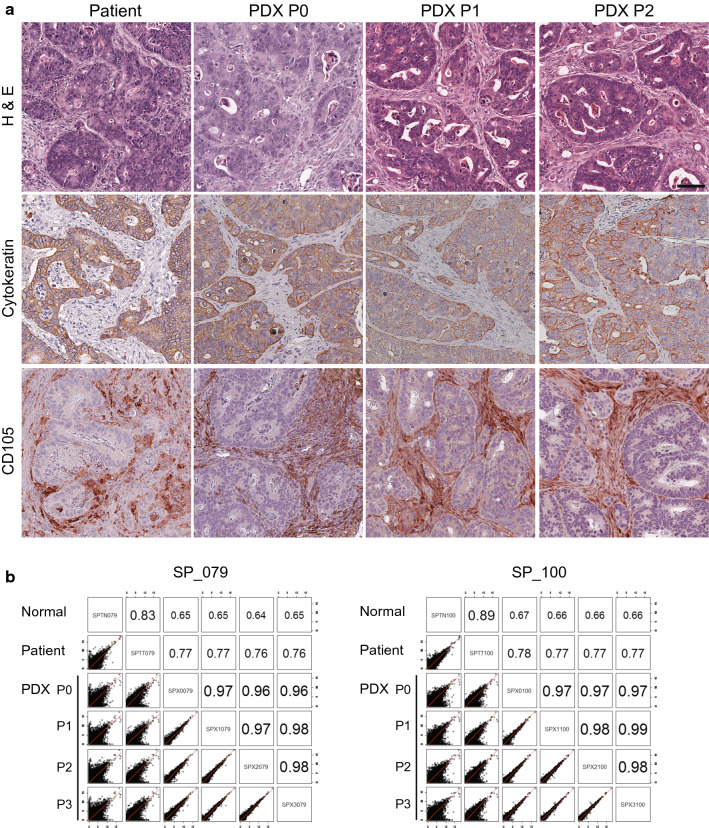
Pathological and genetic concordance of PDX tumors during the passages compared with matched parental patient tumors. **a** Representative images of H&E-stained and IHC-stained tumor samples from the patients with LUSC and PDX passages 0, 1, and 2. Brown indicates pan-cytokeratin-positive or CD105-positive staining. Scale bars, 200 μm. **b** Correlation analysis of transcriptome profiles between the patients with LUSC and PDX passages for SP_079 and SP_100 models

For the PDX models to be useful preclinical study tools in biomarker identification, drug screening, and treatment development [29], retention of their characteristics at the pathological and molecular levels during the PDX passages should be required. Firstly, the authors checked for any pathological changes occurring during the serial passages of the tumor samples from the PDX models compared with the tumor samples from the patients and PDX models at their previous passages (Fig. 5a). Histological images from PDX passage 0 to PDX passage 2 remained the same as those from squamous cell carcinoma. Pan-cytokeratin and CD105, which are human-specific epithelial and endothelial markers, respectively, were invariably expressed in all passages of PDX tumor samples the same as in patient tumor samples, suggesting that PDX models retained the pathological characteristics of their parental patient tumors during the serial passages at least until passage 2.

Secondly, the authors checked for any changes in gene expression patterns in the tumors during PDX passages. The correlation coefficient was over 0.97 between PDX passage 0 and PDX passage 3; however, the correlation coefficient was around 0.77 between the patient and PDX passage 0, which can be interpreted that some difference in gene expression was observed between the tumors in the patient and PDX passage 0 most probably due to the change in tumor host; whereas, the majority of gene expression patterns were found to be considerably preserved in subsequent passages in the same NSG mouse backgrounds (Fig. 5b). The authors also found that expression patterns of DEGs were maintained during the PDX passages as well (Fig. 3b). Taken together, the findings of this study indicated that general pathological and genomic features of the parental patient tumors were well retained during the PDX passages except for the initial shift in gene expression profiles at PDX passage 0 because of the tumor host change.

## Discussion

LUSC is the second most common cancer among NSCLC and is difficult to treat in many cases because it is frequently detected at the advanced stage. LUSC is strongly associated with smoking and is usually accompanied by other comorbidities [30]. These characteristics of LUSC result in poor prognosis despite curative surgical resection followed by adjuvant therapy. The current first-line standard treatment of LUSC is platinum-doublet chemotherapy, while the use of second-line chemotherapy is very limited. Even though molecularly targeted drugs such as anti-EGFR agents and anti-VEGFR2 antibody have been approved recently as a second-line therapy [31], patients with LUSC do not show any significant responses to these drugs due to the lack of dominant driver mutations [12, 32]. Increasing genomic information of LUSC provides us with several promising molecular targets including the *FGFR* gene [33] and their targeted drug candidates. Therefore, preclinical models are still urgently needed to validate the molecularly targeted drugs and to develop effective therapeutic strategies such as combination therapies including immunotherapy.

PDX models have been extensively studied for its potential value as preclinical assessment tools because they maintain the genetic and histological characteristics and intra-tumor heterogeneity and tumor microenvironment of the parental patient tumors at the early passages [29]. Many studies demonstrated that PDX models for various cancer types were useful in discovering biomarkers, identifying therapeutic molecular targets, and evaluating drug responses and treatment regimens [16, 17, 19, 34]. On the other hand, as co-clinical or preclinical study tools, PDX models still are found to have several technical considerations as follows:

Firstly, the authors found the varying degrees of tumor growth and time required for the initial successful engraftments. Tumor growth rates were not found to be uniform in the same NSG mice background, even if the tumor fragments were originated from the same parental patient tumor. The LUSC PDX models of this study showed that the time needed for the initial tumor engraftment ranged between 0.3 and 8.7 months depending upon the patient tumors, and the success rate of initial tumor engraftment determined by the ability of the engrafted tumor to grow up to 800 mm^3^ was not over 45%. Even though the success rate of established PDX models should be based on the result after passage 3 at least, taking into consideration the possibility that occasional models might lead to the eventual failure in tumor growth at the subsequent passages, the authors have experienced that tumor engraftment success rate is over 90% from passage 1 through passage 3 (Additional file 6. Fig. S1). This suggested that the success rate of tumor engraftment was most likely determined by the initial tumor engraftment, and the subsequent passages did not substantially affect the rate, which allows us to consider that the PDX models of this study seem to maintain their stability that is acceptable for various preclinical applications.

The PDX model that needs considerably longer time for initial tumor engraftment would limit its usefulness as a co-clinical study model for advanced LUSC because timely availability is one of the critical requirements for the purpose. However, successfully engrafted PDX models of resectable early-stage LUSC could be used as a co-clinical study model for patients with early-stage LUSC who later would be found to need various drug treatments. These PDX modes would still be useful for drug development or treatment assessment in patients with LUSC with similar genetic backgrounds.

Secondly, the authors found an occasional pathological discrepancy between the tumor samples from patients and the PDX models, such as the XALD phenotype, which occurred at the first stage of tumor engraftment or very rarely at the later PDX passage (one case in this study; data not shown). Several research groups reported that XALD took up a large portion of PDX models [35, 36]. It is still unclear whether EBV would be the sole source of aetiological agents carried over to NSG mice or some other factors would be involved in XADL incidence. In addition, the authors observed that the frequency of XALD incidence was 19.5% among the initial successful engraftments and 12% among the total trials, and could not find any obvious correlation of the XALD phenotype with the shorter time required for the initial successful tumor engraftment.

Thirdly, the authors found occasional genetic inconsistencies between the tumor samples from the patients and PDX models, sometimes during the PDX passages. Ben-David et al. suggested that the genetic alterations between the patient and PDX models were observed because clonal evolution and clonal selection take place either when PDX models are established in different hosts or when PDX models are passaged in the same genetic background of NSG mice [20].

To improve the success rate of initial engraftment, the authors might as well consider a slightly different approach such as the use of orthotopic PDX models [37] presumably because the low success rate of initial tumor engraftment could be caused by the heterotopic interactions of the tumor samples from the patients with the mouse subcutaneous environment, not by the patients’ clinical parameters as shown in Table 1. And to lower the XALD incidence, rituximab treatment to the tumor fragments from the patients prior to xenotransplantation could be employed [35, 36]. Nevertheless, many studies demonstrated that PDX models reproduced the genomic heterogeneity of their parental patient tumors and were considered useful as preclinical study models for improving cancer treatment [17, 38, 39]. Darpkin et al. and Kim et al. demonstrated that PDX models can be applied to co-clinical trials of drug efficacy to optimize the clinical dosage by monitoring the drug response before clinical application [19, 22]. Other groups also showed that the combination of treatments of FGFR inhibitors and conventional chemotherapy could enhance the drug response of patients with lung cancer using PDX models [18, 40–42]. In this context, the LUSC PDX models with *FGFR3* or *BRAF* V600E mutation in this study, as a practical example, should be useful as preclinical study models to test targeted drugs such as dabrafenib [43, 44] and as co-clinical study models to develop a combination strategy [45] as an approach for personalized precision medicine.

## Conclusion

The authors have developed 62 LUSC PDX models that retained the pathological and genomic features of their parental patient tumors, which could be used as tools in preclinical and co-clinical studies.

## Supplementary information

**Additional file 1: Table S1.** The clinical chracteristics of LUSC patients.

S**Additional file 2: Table S2.** Primary antibodies used in the study.

**Additional file 3: Table S3.** Clinical characteristics between XALD and successful PDX engraftment.

**Additional file 4: Table S4.** The freqency of somatic mutations in LUSC patients and PDX models.

**Additional file 5: Table S5.** Somatic mutations and Differently expression genes (DEGs) in LUSC patients and PDX models.

**Additional file 6: Figure S1.** Rate of successful engraftment along the LUSC PDX passages.

## Data Availability

The datasets used and/or analyzed during the current study are available from the corresponding author on reasonable request.

## Abbreviations

LUSC: Lung squamous cell carcinoma
PDX: Patient-derived xenograft
IRB: Institutional review board
NSCLC: Non-small cell lung cancer
SCLC: Small cell lung cancer
LUAD: Lung adenocarcinoma
OS: Overall survival
RFS: Relapse-free survival
NGS: Next generation sequencing
FFPE: Formalin-fixed paraffin-embedded
WES: Whole exome sequencing
WTS: Whole transcriptome sequencing
H&E: Haematoxylin and eosin
IHC: Immunohistochemistry
EBV: Epstein–Barr virus
EBER: EBV-encoded small RNA
XALD: Xenograft-associated lymphoproliferative diseases
CI: Confidence interval
