# Supplementary material for: PDX models of human lung squamous cell carcinoma: consideration of factors in preclinical and co-clinical applications
Source: J Transl Med. 2020 Aug 6;18:307. doi: 10.1186/s12967-020-02473-y (PMC7409653; doi:10.1186/s12967-020-02473-y)
Supplement: Supplementary file 1 — Additional file 1: Table S1. The clinical chracteristics of LUSC patients. [file 12967_2020_2473_MOESM1_ESM.pdf]

Supplemental Table 1. The clinical chracteristics of LUSC patients

	N=139
Age (years)	
Average	66 (39- 82)
Gender	
Male	133
Female	6
Smoking status	
Former or current	137
Never	2
Preoperative Chemotherapy	
Yes	16
No	123
pTNM stage (7th)	
I	46
II	54
III	37
IV	2
Tumor size	
<3cm	38
3< <5	48
5< <7	43
7>	10
Differentiation	
Well	2
Moderate	105
Poor	31
Recurrence	
Yes	28
No	111
Vascular invasion	
Yes	16
No	123
Perineural invasion	
Yes	14
No	125
Lymphatic invasion	
Yes	43
No	96
Visceral pleural invasion	
PL0	107
PL1	3
PL2	12
PL3	15
