# Supplementary material for: PDX models of human lung squamous cell carcinoma: consideration of factors in preclinical and co-clinical applications
Source: J Transl Med. 2020 Aug 6;18:307. doi: 10.1186/s12967-020-02473-y (PMC7409653; doi:10.1186/s12967-020-02473-y)
Supplement: Supplementary file 2 — SAdditional file 2: Table S2. Primary antibodies used in the study. [file 12967_2020_2473_MOESM2_ESM.pdf]

Supplemental Table 2. Primary antibodies used in the study.

**Antibody**	**Vendor**	**Cat. no**	**Dilution**
CK5	Novocastra	XM26	1:50
p63	Dako	M7317	1:300
TTF-1	Dako	M3575	1:50
Pan-cytokeratin	Dako	M3515	1:50
CD105	Santa Cruz	sc 376381	1:100
