# Supplementary material for: PDX models of human lung squamous cell carcinoma: consideration of factors in preclinical and co-clinical applications
Source: J Transl Med. 2020 Aug 6;18:307. doi: 10.1186/s12967-020-02473-y (PMC7409653; doi:10.1186/s12967-020-02473-y)
Supplement: Supplementary file 3 — Additional file 3: Table S3. Clinical characteristics between XALD and successful PDX engraftment. [file 12967_2020_2473_MOESM3_ESM.pdf]

Supplemental Table 3. Clinical characteristics between XALD and successful PDX engraftment

N=78	Growth		p-value
	Success (62)	XALD (15)	
Age(years)			0.634
Mean	66.4±6.8	65.0±11.2	
Gender			
Male	60 (96.8%)	16 (100.0%)	1.000
Female	2 (3.2%)	0 (0.0%)	
Smoking status			
Former or current	61 (98.4%)	16 (100.0%)	1.000
Never	1 (1.6%)	0 (0.0%)	
Preoperative Chemotherapy			0.271
Yes	3 (4.8%)	2 (12.5%)	
No	59 (95.2%)	14 (87.5%)	
pTNM stage (7th)			
I	18 (29.0%)	7 (43.8%)	
II	29 (46.8%)	5 (31.3%)	0.334
III	14 (22.6%)	3 (18.8%)	
IV	1 (1.6%)	1 (6.3%)	
Tumor size			
<3cm	13 (21.0%)	4 (25.0%)	
3< <5	21 (33.9%)	7 (43.8%)	0.601
5< <7	21 (33.9%)	5 (31.3%)	
7<	7 (11.3%)	0 (0.0%)	
Differentiation			
Well	1 (1.6)	0 (0.0%)	1.000
Moderate	47 (75.8)	12 (75.0%)	
Poor	14 (22.6)	4 (25.0%)	
Recurrence			
Yes	14 (22.6%)	3 (18.8%)	1.000
No	48 (77.4%)	13 (81.3%)	
