# Supplementary material for: PDX models of human lung squamous cell carcinoma: consideration of factors in preclinical and co-clinical applications
Source: J Transl Med. 2020 Aug 6;18:307. doi: 10.1186/s12967-020-02473-y (PMC7409653; doi:10.1186/s12967-020-02473-y)
Supplement: Supplementary file 4 — Additional file 4: Table S4. The freqency of somatic mutations in LUSC patients and PDX models. [file 12967_2020_2473_MOESM4_ESM.pdf]

Supplemental Table 4. The freqency of somatic mutations in LUSC patients and PDX models

	**LUSC patient**			**LUSC PDX models**		
ID	Gene	Mutation	Frequency	Gene	Mutation	Frequency
DP_032	TP53	H179R	0.478261	TP53	H179R	1
	PTEN	A148fs	0.371681	PTEN	A148fs	0.454936
	EP300	C1247Y	0.0406504	CDKN2A	R52W	0.5
DP_040	HRAS	G13V	0.388889	HRAS	G13V	1
	TP53	Y236C	0.395349	TP53	Y236C	1
	PTEN	-78fs	0.44	PTEN	-78fs	0.488095
				GNAQ	T96S	0.142857
				GNAQ	M59L	0.08
				U2AF1	G167C	0.08
DP_041	MST1R	S802P	0.0568182	CTNNA2	M672I	0.277778
				FRG1	C159Y	0.0419255
DP_043				OBSCN	D3833Y	0.14486
				NFE2L2	D13H	0.508475
				TP53	S215I	0.947368
DP_085	DDR2	Q365K	0.235294	DDR2	Q365K	0.540984
	BRAF	Y472C	0.165354	BRAF	Y472C	0.527273
	BRAF	R146W	0.176923	BRAF	R146W	0.441718
	NF1	W837R	0.287879	NF1	W837R	1
	MUC16	G6037R	0.268398	MUC16	G6037R	0.989529
	MUC16	Q5473H	0.236715	MUC16	Q5473H	0.987342
	GNAS	T225S	0.185185	GNAS	T225S	0.309524
DP_-089	EP300	p.G438V	0.352381	EP300	G438V	0.478873
	AMER1	p.R142K	0.326531	AMER1	R142K	0.851064
	RB1	p.I425fs	0.426667	RB1	I425fs	0.471947
				IGF2R	D1389A	0.397959
DP_091	CTNNA2	R136H	0.5	CTNNA2	R136H	0.578947
	CTNNA2	L872M	0.582278	CTNNA2	L872M	0.557047
	ERBB4	I119V	0.245902	ERBB4	I119V	0.25
	SOX2	M4I	0.5	SOX2	M4I	0.9
	PDGFRA	N471S	0.438596	PDGFRA	N471S	0.438596
	JAK2	R1127S	0.823529	JAK2	R1127S	0.823529
	GNAS	P707T	0.409091	GNAS	P707T	0.392857
	NF1	S1524fs	0.472678	NF1	S1524fs	0.47191
	EYS	S658R	0.850394	EYS	S658R	1
	MUC16	S10327F	0.788462	MUC16	S10327F	0.990654
	MUC16	G6924W	0.815249	MUC16	G6924W	0.97493
	MUC16	H3782Q	0.870504	MUC16	H3782Q	0.981707
	MUC16	G274V	0.818966	MUC16	G274V	1
	MUC16	V2144F	0.813084	MUC16	V2144F	0.966667
	OBSCN	G3952V	0.41573	OBSCN	R2985L	0.0666667
	MUC16	S8133R	0.0362319	OBSCN	G3952V	0.366071
	ALK	H344Y	0.118012	ALK	H344Y	0.606838
	ATR	Q1293P	0.0547945	ATR	Q1293P	0.192118
	PIK3CA	R88Q	0.111111	PIK3CA	R88Q	0.237226
