# Supplementary figures and images for: PDX models of human lung squamous cell carcinoma: consideration of factors in preclinical and co-clinical applications
Source: J Transl Med. 2020 Aug 6;18:307. doi: 10.1186/s12967-020-02473-y (PMC7409653; doi:10.1186/s12967-020-02473-y)

Supplemental Figure 1. Rate of successful engraftment along the LUSC PDX passages

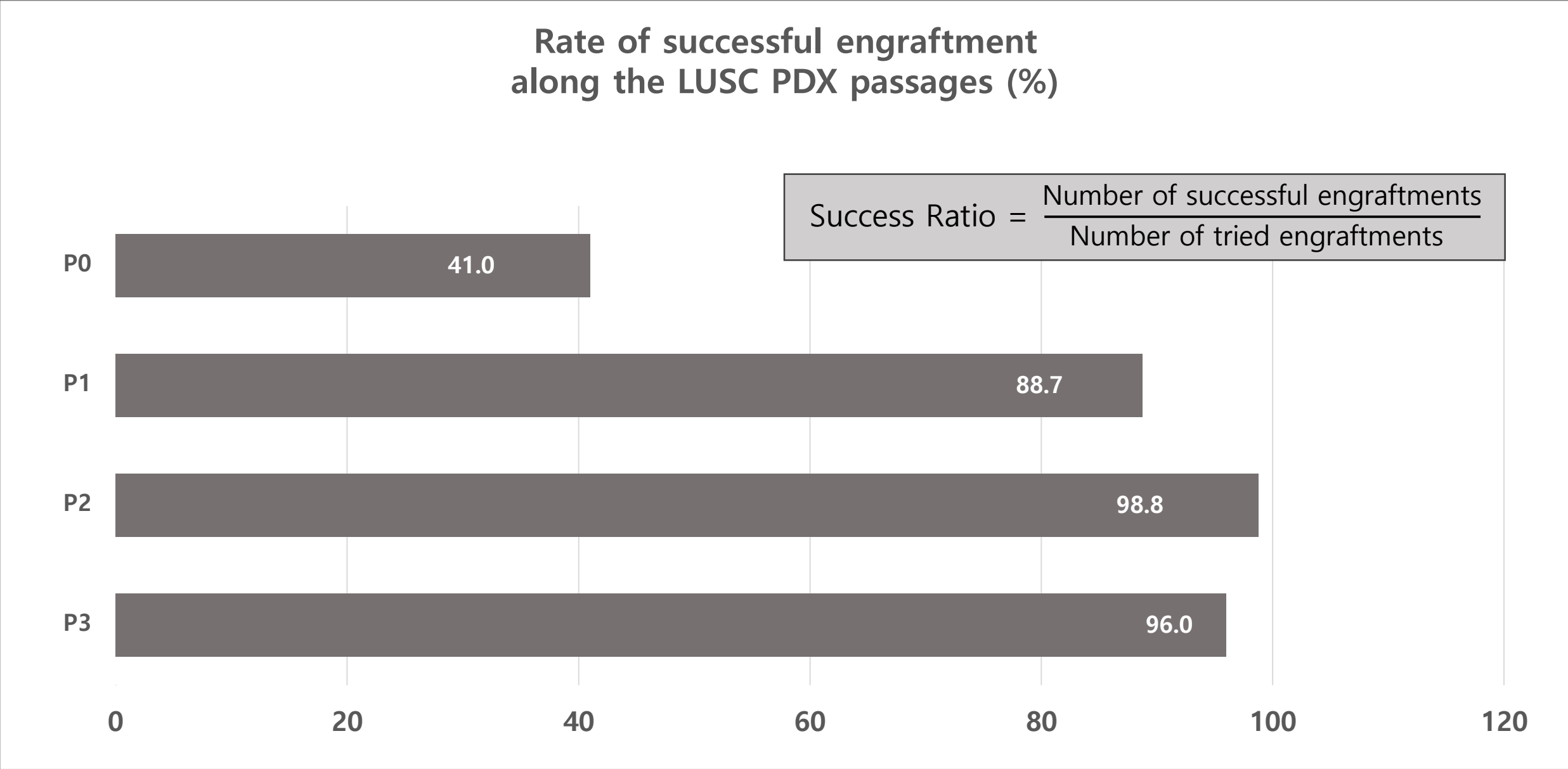

Supplement: Supplementary file 6 — Additional file 6: Figure S1. Rate of successful engraftment along the LUSC PDX passages. [file 12967_2020_2473_MOESM6_ESM.pdf]
