# Supplementary material for: PDX models of human lung squamous cell carcinoma: consideration of factors in preclinical and co-clinical applications
Source: J Transl Med. 2020 Aug 6;18:307. doi: 10.1186/s12967-020-02473-y (PMC7409653; doi:10.1186/s12967-020-02473-y)
Supplement: Supplementary file 5 — Additional file 5: Table S5. Somatic mutations and Differently expression genes (DEGs) in LUSC patients and PDX models. [file 12967_2020_2473_MOESM5_ESM.pdf]

Supplemental Table 5. Somatic mutations and Differently expression genes (DEGs) in LUSC patients and PDX models.

**Somatic Mutations**

|Sample|ABCA1|AKT1|ALK|AMER1|APC|ARID1A|ATR|ATRX|BAI3|BRAF|CDKN2A|CREBBP|CTNNA2|CUL3|DDR2|EP300|ERBB4|EYS|FBXW7|FGFR2|FLT1|FLT3|FRG1|GNAQ|GNAS|GRM8|HLA-A|HRAS|IGF1R|IGF2R|JAK2|KDR|KEAP1|LTK|MET|MST1R|MUC16|MYH1|NF1|NFE2L2|NOTCH1|NOTCH2|OBSCN|PDGFRA|PIK3CA|PRG4|PTEN|RB1|ROS1|SMO|SOX2|STK11|TNFAIP3|TP53|TRRAP|TSC1|TSC2|U2AF1|ZEB2|
|---|---|---|---|---|---|---|---|---|---|---|---|---|---|---|---|---|---|---|---|---|---|---|---|---|---|---|---|---|---|---|---|---|---|---|---|---|---|---|---|---|---|---|---|---|---|---|---|---|---|---|---|---|---|---|---|---|---|---|---|
|DP_032|||||||||||||||1|||||||||||||||||||||||||||||||2|||||||1||||||
||||||||||||1||||||||||||||||||||||||||||||||||||||2|||||||1||||||
|DP_040|||||||||||||||||||||||||||1|||||||||||||||2|||||||1||||||
||||||||||||||||||||||||2||||1|||||||||||||||||2|||||||1||||1||
|DP_041|||||||||||||||||||||||||||||||||||1||||||||||||||||||||||||
|||||||||||||||1||||||||||1||||||||||||||||||||||||||||||||||||||||
|DP_043||||||||||||||||||||||||||||||||||||||||||||||||||||||||||||
|||||||||||||||||||||||||||||||||||||||||1|||1|||||||||||1||||||
|DP_085||||||||||2|||||1|||||||||||1|||||||||||||||2||1|||||||||||||||||||||
||||||||||||2|||||1|||||||||||1|||||||||||||||2||1|||||||||||||||||||||
|DP_089||||1|||||||||||||1|||||||||||||||||||||||||||||||||||||||2|||||||||||||
|||||1|||||||||||||1|||||||||||||||||1||||||||||||||||||||2|||||||||||||
|DP_091||||||||||||||2||||1|1|||||||1|||||||1||||||6||2||||1|1|||||||1|||||||||
||||||||||||||||2||||1|1|||||||1|||||||1||||||5||2||||2|1|||||||1|||||||||
|DP_099||||1||||1|||||1|||||||||||||||||||||||||||||||1|||2|||1||1||||1|||||||||||
||||1||||1|||||1||||||||||||||||||||||||||||||||1||2|||||1|4|||1|||||1||||||
|DP_118|||||||||1|||||||||||1|||||||||||||||||1||||4|||||||||||||||||||||||||||||||
||||||||||1||||||1||||||1||||||1||||||||||||||1||||1||||||||||||||||||||||||||||||
|SP_073||||||||||||||||||1|||||||||||||||||||||||||||||||||||||||||||||
||||||||||||||||||||1|||||||||||||||||||||||||||||||||||||||||||||
|SP_079||||||||||1|||||||||||||||1|||||||||||||||||||||||||||||||||||||1||||||
||||||||||||1|||||||||||||||1|||||||||||||||||||||||||||||||||||||1||||||
|SP_100||||||||||1||||||1|||||||||||1|||||||||||||||||||||||||||||||||||2||||||
||||||||||||1||||||1|||||||||||1|||||||||||||||||||||||||||||||||||2||||||
|SP_186|||||||||||||||||||||||||||||||||1||||||||||||||||||||||||||||
|||||||||||||||||||||||||||||||||||1||||||||||||||||||||||||||||
|SP_190|||||||||||||||||||||||||||||||||||||1|1||||||||||||||||||||||1||||||
||||||||||||||||||||||||||||||||||||||||||||||||||||||||||||||
|SP_205||||||||||||||||||||||||||||||||||||||||||||||||||||||||||||
||||||||||||||||||||||||||||||||||||||||||||||||||||||||||||||
|SP_207||||||||1|1||||||||||||||||||||||||||||||||||||||||||||||||||||1||||||
||||||||||||||||||||||||||||||||||||||||||||||||||||||||||||||
|SP_212|||||||||||||||||||||||||||||||||||||||||||||||||||||1||||1||
|||||||||||||||||||||||||||||||||||||||||||||||||||||||1||||||
|SP_231|||||||||||||||||||||||1||||1||||||||1||1||1|1|||||||||1||||||1||||||||
|||||||||||||||||||||||||1||||1||||||||1||1||2|1|||||1|||1||||1||1||||||||
|SP_252||||||||||||||||||||||||||||||||||||||||||||||||||||||||||||
||2|||||||1||||||||||1|||||||1||||6||||||||||2|21||||||2|||3|||1||||||1||||
|SP_271|||1||||||||||1||||||||||||||||||||||||||||||||||||||||||||||1||||||
||||||||1||||||||||||||||||||||||1||||||||||||||||||||||||||||1||||
|SP_302|||||||||||||||||||||||||||||||||||||||||||||||1||||||||1||||||
||||||||||||||||||||||||||||||||||||||||||||||||||||||||||||||
|SP_318|||||||||||||||||||1||||||||2||||||||||||2||1||||||1|||||||2||||2|1|||||
|||||||1||||||||||||1||||||1|||2||||||||||||1||1||||||1|||||||2||||2|1|||||
|SP_364||||||||||||||||||||||||||||||||||||||||||||||||||||||||||1|||
||||||||||||||||||||||||||||||||||||||||3||||||||||||||||||||1|||
|SP_366|1||||||||||||||||||||||||1|||||||||1|||||||||1|||||||||||||1||||||
||1||||||||||||||||||||||||1|||||||||1|||||||||1|||||||||||||1||||||
|SP_371||||||1|||||1||||||||||||||||||||||||||||||||||||||||1||||||||||||
||||||||||||||||||||||||||||||||||||||||||||||||||||||||||||||
|SP_372|||||||||||1||||||||||||||||||||||||||||1||||||||||||||||||||2||||||
|||||||||||||1||||||||||||||||||||||||||||||2|||||||||||||||||||2||||||
|SP_377||1|||||||1|||||||||||||||||||||||||||||||2||||||||||||||||||||1||||||
||||1||||||||||||||||||||||||||||||||||||||1||||||||||||||||||||1||||||
|SP_402|||1||||||||||||||||1||||||3|||1||||||||||||2|1|||||||1|||||||||||||||
|||||1||||||||||||||||1||||||1|1||1||||||||||||3|1|||||||1|||||||||||||||
|SP_409||||||||||||||||||||||||||||||||1|||||1||||||||1|||||||||||||||||
|||||||||||||||||||||||||1|1||||||||||1|||||2|||||||||1|||||||||||||||
|SP_410||||||||||||||||||||||||||||||||2||||||||||||||||||||||||||1||||||
|||||||||||||||||||||||||||||||||||2|||||1|||||||||||||||||||||1||||||
|SP_415|||||2||||||2|1||||||||||||||||||||||||||||||||||2|||||||||||||1||||||
|||||2|2||||||2|1||||||||||1|||||||||||||||||||||||||||2|||||||||||||1||||||
|SP_420|||||||||||||||||||||||||||||||||||||1||||1||||||||||||||1||||||
|||||||||||||||||||||||||||||1||||||||||1||||1||||||||||||||1||||||
|SP_434|||||||||||||||||||||||||||||||||1||||1|1||||1|||||||||||||||||2||||||
|||||||||||||||||||||||||||||1||||1|||||1||||1|||||||||||||||||2||||||
|SP_448|||||||||||||||||||||||||||||||||||||1|||||||||||||||||||||||||
|||||||||||||||1|||||||||||2||||||||||||||||1|||||||||2|||||||||||1|||
|SP_451||||||||||||||||||||||||1||||1||||||||||||||||||||2|||1||||||||||||||
||||||||||||||||||1|||||||1||||3|||||||||||||||||||||2|||1||||||||||||||
|SP_454||||||||||||||||||||||||||||1||||||||||||2|||||||||||||||||||1|||||||
|||||||||||||||||||||||1|||||1|||||||||||||2||2|||||||||||||||||1|2||||||
|SP_473||||||||||||||||||||||||1|||||||||||||1||||||||||||||||||||1||||||||||||
|||||||||||||1|||||||||||1|||||||||||||1||||||||||||||||||||1||||||||||||
|SP_480||||||||||||||||||||||||||||||||||||||||3||||||||||||||||||1|||1||1|
||||||||||||||||||||||||||||||||||||||||1||||||||||||||||||1|||1||1|
|SP_493||||||||||||||||||||1|||||||||||||||||1|||1||||||||||||||||||||
||||||||||||||||||||||||||||||||||||||||||||||||||||||||||||||
|SP_513||||||||||||||||||||||1|||1|||1|||||||1||||||||||||||||1|||||||||||||||||
||||||||||||||||||||||||||||||||||||||||||||||||||||||||||||||
|SP_522||||||||||||||||||||||||||||||||||||||||||||||||||||||||||||
||||2||||||||||||||||4|||||1||||||||||||||||||||||||1||1|||||||||2||1||||
|SP_524|||||||||||||||||||||||||||||||||||||||||||||||||||||2||||||
|||||||||||||||||||||||||1||||||||||||||1||||||||||||||||||||2||||||
|SP_547||||||||||||||||||||||||||||||||||||||||||||||||||||||||||||
||||||||||1||1||||1|||||||||||||1|||||||||2|||1||||||2|2|||||||||||||
|SP_552||||||||||||||||||||||||||||||||||||||||||||||||||||||||||||
|||||||||||||||1|1|||1||||1|||||1|||||||||||||||||||||||3|||||||||4||||||
|SP_584|||||||||1||||||||||||||1||||||||||||||||||||||||||||||1|||||1||||||
|||||||1|||1|||||||||||||||||||||||||||||||||||||||||||1|||||1||||||

**Differentially expressed genes (DEGs)**

|Sample|ABCA1|ABL2|AKT1|AKT3|ALK|AMER1|APC|AR|ARID1A|ATR|ATRX|BAI3|BCL2L1|CDK6|CDKN2A|CREBBP|CTCF|CTNNA2|CTNNB1|DDR1|DDR2|EGFR|EP300|EPHB3|ERBB2|ERBB4|FBXW7|FGFR1|FGFR2|FGFR3|FLT1|FLT3|FOXP1|GNAQ|GNAS|GRM8|HIVEP3|HLA-A|HRAS|IGF1R|
|---|---|---|---|---|---|---|---|---|---|---|---|---|---|---|---|---|---|---|---|---|---|---|---|---|---|---|---|---|---|---|---|---|---|---|---|---|---|---|---|---|
|DP_032|||||||||||||||||||||||||||||||||||||||||
||||||||||||||||||||||||||||||||||||||||||
|DP_040|||||||||||||||||||||||||||||||||||||||||
||||||||||||||||||||||||||||||||||||||||||
|DP_041|||||||||||||||||||||||||||||||||||||||||
||||||||||||||2|5|||||3|-5|2||4||-10|||||-4||-2|||||-2|3||
|DP_043|||||||||||||||||||||||||||||||||||||||||
||||||||||||||||||||||||||||||||||||||||||
|DP_085|||||||||||||||||||3||||||||||||||||||||||||2|
|||||-2||||-3||||-2||2||||||2|-4|2||3||-4||-3|2|2|-3||-2|-2|||||3|2|
|DP_089||||||||||||-3|||-2|||-3||||||4||-5|||||3||||||||||||
||||2|||2||||||||||-4|||||2|-4|||3||-2|||2|5|-2||-2|-2||||-3|2||
|DP_091|||||-4|||||||2||||-4|||-4|2|-2|||||||||-4||||||||||||||
||2|-2||-3|-3|2||-4||2|2|-5|2||-11||2|-6|2|-1||3|3|2||||-3|-5|3|-3|||||-4||-2||2|
|DP_099||||-2|||||||||||7|||||2|-2|||2||-5||-3|2|4|||-2||||||||
||-3|||-4|||||-2||||||6|||||2|-8||-2|3||-10||-4||4|-7||-7|-2|||-5|-2|||
|DP_118|||||||||||||||||||||||||||||-2|||||||||||||
||-2|-2||-4|||||||||||5|-2||||2|-5|2||2||||||5|-7||||||-4|-3|||
|SP_073||||||||||||||||-3||||||2|-4|3||||||-2||||||-3||||-2||||
||||||||||||-2|||-4||||||2|-7|3||||||||||||-6||||-3||||
|SP_079||||||||||||||||4||||||||||2||-4|||||||||||||||
||-3|||-2|||||2||||3|4||||||-5|2||4||-9|-2|-3||3|-6|||-2|||||2|2|
|SP_100||||||||||||||||2|3||||||||||||||||||||||||||
||-5|||-6|||||||||||4|4||||||-6||||||-3||-8|-2|-6|||-2||||2|||
|SP_186|||||||||||||||||||||||||||||||||||||||||
||||||||||||||||||||||||||||||||||||||||||
|SP_190|||||||||||||||||||||||||||||||||||||||||
||||||||||||||||||||||||||||||||||||||||||
|SP_205||||||||||||||||2|||||-2||-2||||-5|||-5|-4|||||||||||
||||||||||||||||||||||-3||-4|||-2|-4|||-5|-6|-4|||-2||||2||-2|
|SP_207|||||||||||||||||||||||||||||||||||||||||
||||||4|3||-2||||-7|||3||||2|2|-8|||5||-4|||2||-3||-2||||-2||2||
|SP_212||||-3||||||||-5||2|4|||||||||3||-3|||2|5|-2|||-2||||-3|||
||-3|||-4||2||||||-10||2|4|||||2|-4|||4||-4|||2|6|-6|||-2||||-5|||
|SP_231||||||||||||-4||||||||||-2||||-4||||-3|||||||||||
||-4||||||-4|-2|||||||2|3|||6|||-4|-4||2||-4|||-4||-5|||-3|||-3|-3|||
|SP_252|||||||||||||||||||||||||||||||||||||||||
||||||||||||||||||||||||||||||||||||||||||
|SP_271|||||||||||||||||||||||||||||||||||||||||
||||||||||||||||||||||||||||||||||||||||||
|SP_302||||||||||||-3|||5|||||||||3||-6||||||||||||||2||
|||||-3||||-5||||-9|2|3|4||||||-5||||-2|-10||-3|-5|-4|-2||-4|-5||||2||-3|
|SP_318|||||||||||||||||||||||||||||||||||||||||
||||||||||||||||||||||||||||||||||||||||||
|SP_364|||||||||||||||||||||||||||||||||||||||||
||||||||||||||||||||||||||||||||||||||||||
|SP_366|2|||||3||-2||2||-4||2|-4|||-3||2||3||||-2||-2||2||||||-3|||2||
||||||||||||||||||||||||||||||||||||||||||
|SP_371|||||||||||||||||||||||||||||||||||||||||
||||||||||||||||||||||||||||||||||||||||||
|SP_372||||||||||||||||3||||||-3|||||-3||||||||||3|||||
||-2|||-4||2||-4||||||4|||||3|-6||2|2||-2|-2||2|2|||-2|||||-2|2|2|
|SP_377|||||||||||||||||||||||||||||||||||||||||
||||||||||||||||||||||||||||||||||||||||||
|SP_402|||2|||||||||||2|5||||||||||4||-7||||2|||||||||2||
||||3|||||-3|||||||2|6|||||3|-7|||5||-5||-2|2|3|-6||-2|-2||||-3|2||
|SP_409||2|2|-2||2||-2||2||-3|||4||2|-2|2|3||2||3||-4|2|||4||-3|||2||||4||
|||2|2|-4||3||-3|3|2|2|-10|2||4||2|-4|2|3|-4|2|2|4||-6|||2|5|-4|-9|-2||2|-3|-2||4||
|SP_410||||||||||||||||4||||||||||||2|||||||||||||
||-2|||-6||2||-4|||||||5|||||3|-7|||5|||-2|-3|3||-7|-6|-3||2||-4||||
|SP_415|||||||||||||||||||||||||||||||||||||||||
|||||||3||-3|2|2||-3|||3|||||2|-6|-6|2|3||-5|-2|||||-4||||||-3|-7|||
|SP_420|||||||||||||||||||||||||||||||||||||||||
||||||||||||||||||||||||||||||||||||||||||
|SP_434||||||||||||||||4||||||||||||-3|||2|||||||||||||
||-2|||||2||||||||3|5|||||2|-6|3||2||-4||-2||3|-5||-4|-3||||-2|||
|SP_448||||||||||||||||2||||||||||2||-4||||3|||||||||||
|||||-3|||||||||||2|||||2|-6|3||2||-3||-8||4|-7||-4||||||2||
|SP_451|||||||||||||||||||3||||||||2||-2|||||||||||||||
|||||||2||-3||3||||||||||3|-5|3||2||-6||-2||2|-5||-2||||||2||
|SP_454||||-2||2||-3||||-5||2|4|||||3|-2|2||2||-5||-3||4||||||||-2|3|2|
|||||-3||2||-2||||-9||2|4|||||3|-7|2|2|2||-3||-4||5|-3||-2|-2||||-4|||
|SP_473|||||||||||||||||||||||||||||||||||||||||
||||||||||||||||||||||||||||||||||||||||||
|SP_480||||-2||||||||-4|||3|||||2||||3||-5|||-2|||||||||||||
||-4|||-5||||||||-4|2|2|5|||||2|-5|||2||-7||-5|-2|2|-5||-2|||||-2|||
|SP_493|||||||||||||||||||||||||||||||||||||||||
||||||||||||||||||||||||||||||||||||||||||
|SP_513|||||||||||||||||||||||||||||||||||||||||
||||||||||||||||||||||||||||||||||||||||||
|SP_522|||||||||||||||||||||||||||||||||||||||||
||||||||||||||||||||||||||||||||||||||||||
|SP_524|||||||||||||||||||||||||||||||||||||||||
||||||||||||||||||||||||||||||||||||||||||
|SP_547|-3|||||||-3|||||||2|4|||||2|-4|2||||-2|||2||-6||-3||||-3|-2|2||
||||||||||||||||||||||||||||||||||||||||||
|SP_552|||||||||||||||||||||||||||||||||||||||||
||||||||||||||||||||||||||||||||||||||||||
|SP_584|||||||||||||||||||||||||||||||||||||||||
||||||||||||||||||||||||||||||||||||||||||

|Sample|IGF2R|JAK2|KDR|KEAP1|KIT|KRAS|LTK|MDM2|MET|MST1R|MUC16|MYC|MYH1|NFE2L2|NOTCH1|NOTCH2|OBSCN|PDGFRA|PIK3CA|PRG4|PTEN|RAC1|RHOA|ROS1|RUNX1|RUNX1T1|SMAD4|SMARCA4|SMO|SOX2|STK11|TNFAIP3|TP53|TP63|TRRAP|TSC1|U2AF1|VHL|WHSC1L1|ZEB2|
|---|---|---|---|---|---|---|---|---|---|---|---|---|---|---|---|---|---|---|---|---|---|---|---|---|---|---|---|---|---|---|---|---|---|---|---|---|---|---|---|---|
|DP_032|||||||||||||||||||||||||||||||||||||||||
||||||||||||||||||||||||||||||||||||||||||
|DP_040|||||||||||||||||||||||||||||||||||||||||
||||||||||||||||||||||||||||||||||||||||||
|DP_041|||||||||||||||||||||||||||||||||||||||||
||||-3||-6|||||3||||||||-4||-6||||-2||-5|||||||||7||||||-5|
|DP_043|||||||||||||||||||||||||||||||||||||||||
||||||||||||||||||||||||||||||||||||||||||
|DP_085|||||-2|||||||||||||||||||||-4|||||2|7||||4|||||||
|||-3|-5||-6||||2|||||||||-5||-2||||-5||-2|||3|8||||6||||||-6|
|DP_089|||||-2|||||||||||||||||-5||||-8||||||5||||5|||||||
|||-6|-4|2|-3|||||-3||||3||||-3|2||||||-6|||||-4|8||-5|-3|7||||||-4|
|DP_091||||||2||2||||-4|3||2||||||2|2|2||-5|||2|||||||-2|2||||2||
|||-2|-11||-3|2|2|2|||-9|3||2||2||-4|||2|2||-6||-6|2||2|||-2||-3|2|||2|2|-7|
|DP_099||||-2|2|-4|||||||||||||||-5||||-4||-3||||7|||2|7||||||
|||||-8|3|-8|||||-2||||||-2||-5||-5|-2|||-6|-2|-5|||2|7||-3||6||||||-4|
|DP_118||||||||||-4|||15||||2|||||||-6|||||||||||4|||||||
|||-3|-7|3|-3||||||2|||||||-6||-6|-7|||-9|-3|-4||||8||-4||6||||||-8|
|SP_073|||-2|-4||-4||||||||||||-2||-7||||-4||-3|||||||||3||||||-2|
|||-2|-13||-9||||||||||-2|||-6||-6||||-10||-7||2|||||-2||3||||||-7|
|SP_079||||-2||-3||||||6|||||||||-5||||||-2|||||||||7|||||||
|||-2|-9||-6|||||3|9|||||||-7||-5||||-13||-6||2||||||7||||||-8|
|SP_100||||||||||3|||||||||||||||||-2|||||||||||||||
||||-8|-2|-7|||||4|3|||||||-7||||2||-13||-7|||-10|2||-3|||||||2|-9|
|SP_186|||||||||||||||||||||||||||||||||||||||||
||||||||||||||||||||||||||||||||||||||||||
|SP_190|||||||||||||||||||||||||||||||||||||||||
||||||||||||||||||||||||||||||||||||||||||
|SP_205|||||-3||||||||||||||||-5||||-5|||||||||3|||||||
||||-4||-5||||-6|-3||-3||||||||-4||||-8|||||||||||||||||
|SP_207|||||||||||||||||||||||||||||||||||||||||
||2|-3|-8||-6|||2||-10|-5|||2||||-5|3|-5||2||||-6|||3|5||-2|3|5||||||-9|
|SP_212||||-2||-3||-6|||||||-2||||||||-4||-2|||3|4||-3||5||||||-3|
|||||-3||-5||-6|||||||-2|||-5||||-2|-5||-5|||3|5||-4||6||||||-7|
|SP_231||||-2||-2||||-2||||||||||-4||||-9||||||4|||||||||||
|||||-4|2|-3||||-2|||2|||-2|||-5||-4||||-9||-7||2|2|6||||||||||-6|
|SP_252|||||||||||||||||||||||||||||||||||||||||
||||||||||||||||||||||||||||||||||||||||||
|SP_271|||||||||||||||||||||||||||||||||||||||||
||||||||||||||||||||||||||||||||||||||||||
|SP_302|||||-2||||||||||||||||-5||||-3||-2||||2||||5|||||||
||||-12||-8|||2|||||||||-2|-4|-4||-5||||-8||-8|||-5|||2||2||||||-3|
|SP_318|||||||||||||||||||||||||||||||||||||||||
||||||||||||||||||||||||||||||||||||||||||
|SP_364|||||||||||||||||||||||||||||||||||||||||
||||||||||||||||||||||||||||||||||||||||||
|SP_366||||3|-2|||||||-2|||4||||||-3||2||||-2|||4|7|2|-2||6||||||-2|
||||||||||||||||||||||||||||||||||||||||||
|SP_371|||||||||||||||||||||||||||||||||||||||||
||||||||||||||||||||||||||||||||||||||||||
|SP_372||||-2|||||||||||2|||||||||||-4||-2|||2|7||||4||||||-2|
|||-3|-5|2|-3||3|||2||2||2||||||||2||-6||-6||2|2|8||-2||5||||||-8|
|SP_377|||||||||||||||||||||||||||||||||||||||||
||||||||||||||||||||||||||||||||||||||||||
|SP_402|||||-3|||||-2||||2||||||-4||||-7||||||6||||6|||||||
||||-7||-4|||||-5||||3|||2|-3||-5||||-6||-7|||2|8||||8||||||-5|
|SP_409||||3||||||||-4|2||2|||||2|||2||-7|||||2|6||||7|||2||3|-2|
||||-3|3|-2|2||1||||-2|2||2||||-2|2|-3||2||-6||-5||2|2|6|2||2|6||2|||3|-4|
|SP_410||||||||||||||||||||||||||||||2|9||||5|||||||
||||-12|2|-6||||||6|||||||-4|2||||||-13||-7|||4|10||-4|2|6||||||-6|
|SP_415|||||-3||||||||||||||||||||||||||||||||||||||
|||-4|-4||-7|2|||||||||||||-4|||-2|||-10||-2||3||8||-4|2|||||||-8|
|SP_420|||||||||||||||||||||||||||||||||||||||||
||||||||||||||||||||||||||||||||||||||||||
|SP_434|||||-3||||||||||||||||||||-3||||||8||||6|||||||
|||-4|-7|2|-7||||3|||2||||||-4|||||||-5||-4||2|3|7||-2||8||||||-7|
|SP_448|||||-3||||||||||||||||||||-4||||||-4||||2|||||||
|||-2|-12||-8||||||||||||||-5|||||||||-3||||-3||-2||4||||||-8|
|SP_451||||-3||-2||-5|||||||||||-2||||||-2|||||3|6||||8|||||||
||||-12|2|-5|2|-5||2||||||||||-3||||||-4||-6|||4|8||||8||||||-6|
|SP_454||||-2||-2||||2||4|2||||||||-5||2||-3||-4||2||7||||7||||||-2|
||||-11|2|-5||||||5|||||||-3||-4||2||-3||-6||2|2|7||||7||||||-8|
|SP_473|||||||||||||||||||||||||||||||||||||||||
||||||||||||||||||||||||||||||||||||||||||
|SP_480|||||-3|||||2||||||||||-7||||-4||||||3||||6|||||||
|||-2|-9||-6|||||3|4|2||||||-6||-5||||-9||-5||||5||-2|2|6||||||-6|
|SP_493|||||||||||||||||||||||||||||||||||||||||
||||||||||||||||||||||||||||||||||||||||||
|SP_513|||||||||||||||||||||||||||||||||||||||||
||||||||||||||||||||||||||||||||||||||||||
|SP_522|||||||||||||||||||||||||||||||||||||||||
||||||||||||||||||||||||||||||||||||||||||
|SP_524|||||||||||||||||||||||||||||||||||||||||
||||||||||||||||||||||||||||||||||||||||||
|SP_547||-2|-6|2|-5|||||-3||2||||||-2||-6||||-7||-6|||3|5||||4||||||-3|
||||||||||||||||||||||||||||||||||||||||||
|SP_552|||||||||||||||||||||||||||||||||||||||||
||||||||||||||||||||||||||||||||||||||||||
|SP_584|||||||||||||||||||||||||||||||||||||||||
||||||||||||||||||||||||||||||||||||||||||

The PDX models were placed on the y−axis to display the information of somatic mutations and DEGs. In the 2−row for each PDX model, the yellow box at the top row is for the patient data, the green box at the bottom row for PDX data, the colorless box for no data available. In each column of the somatic mutation section, the number of mutations for each gene was indicated, and in each column of the DEG section, the expression level for each gene was indicated by number and color (red, upregulation and blue, downregulation).
